# Risk factors for delayed negative conversion of SARS-CoV-2 in patients with COVID-19 pneumonia: a retrospective cohort study

**DOI:** 10.1017/S0950268820002940

**Published:** 2020-12-01

**Authors:** Pingzheng Mo, Liping Deng, Xiaoping Liu, Shicheng Gao, Ke Liang, Mingqi Luo, Tielong Chen, Shihui Song, Zhiyong Ma, Xiaoping Chen, Junli Fan, Fan Wang, Yong Xiong, Yongxi Zhang

**Affiliations:** 1 Department of Infectious Diseases, Zhongnan Hospital of Wuhan University, Wuhan 430071, Hubei, China; 2 Department of Urology, Zhongnan Hospital of Wuhan University, Wuhan 430071, Hubei, China; 3 Department of Laboratory Medicine, Zhongnan Hospital of Wuhan University, Wuhan 430071, Hubei, China; 4 Department of Gastroenterology, Zhongnan Hospital of Wuhan University, Wuhan 430071, Hubei, China

**Keywords:** COVID-19, negative conversion, risk factors, SARS-CoV-2

## Abstract

The epidemic of coronavirus disease 2019 (COVID-19) began in China and had spread rapidly to many other countries. This study aimed to identify risk factors associated with delayed negative conversion of SARS-CoV-2 in COVID-19 patients. In this retrospective single-centre study, we included 169 consecutive patients with confirmed COVID-19 in Zhongnan Hospital of Wuhan University from 15th January to 2nd March. The cases were divided into two groups according to the median time of SARS-CoV-2 negative conversion. The differences between groups were compared. In total, 169 patients had a median virus negative conversion time of 18 days (interquartile range: 11–25) from symptom onset. Compared with the patients with short-term negative conversion, those with long-term conversion had an older age, higher incidence of comorbidities, chief complaints of cough and chest distress/breath shortness and severer illness on admission, higher level of leucocytes, neutrophils, aspartate aminotransferase, creatine kinase and erythrocyte sedimentation rate (ESR), lower level of CD3^+^CD4^+^ lymphocytes and albumin and more likely to receive mechanical ventilation. In multivariate analysis, cough, leucocytes, neutrophils and ESR were positively correlated with delayed virus negative conversion, and CD3^+^CD4^+^ lymphocytes were negatively correlated. The integrated indicator of leucocytes, neutrophils and CD3^+^CD4^+^ lymphocytes showed a good performance in predicting the negative conversion within 2 weeks (area under ROC curve (AUC) = 0.815), 3 weeks (AUC = 0.804), 4 weeks (AUC = 0.812) and 5 weeks (AUC = 0.786). In conclusion, longer quarantine periods might be more justified for COVID-19 patients with cough, higher levels of leucocytes, neutrophils and ESR and lower levels of CD3^+^CD4^+^ lymphocytes.

## Introduction

Since December 2019, the epidemic of coronavirus disease 2019 (COVID-19), caused by severe acute respiratory syndrome coronavirus 2 (SARS-CoV-2) began in China and had spread rapidly to many other countries [1–6]. The whole world had faced the unusual challenge of the high SARS-CoV-2 infectivity.

It is important to control the outbreak of COVID-19 rapidly and effectively. Extensive measures have been implemented that include early diagnoses, isolation and antiviral treatments. There are currently more than 180 vaccines at various stages of development, many of which have moved into phase III trials [7]. However, there is no vaccine and effective antiviral drug available yet. In the absence of vaccines and effective treatments, the best way to deal with the SARS-CoV-2 epidemic is to control the sources of infection. Currently, the COVID-19 patients remain as the main source of infection [8]. Patients in incubation and recovery period may also be infectious. The previous study showed that the incubation period was 4–12 days. [9]. Another study suggested that median duration of SARS-CoV-2 shedding was 20 days (interquartile range (IQR): 17–24) in surviving patients [10]. The existence of long-term virus carriers was the difficulty of controlling the epidemic situation. To our knowledge, no previous studies had been carried out among COVID-19 patients with delayed negative conversion of SARS-CoV-2. In this study, we aimed to explore the risk factors for delayed negative conversion of SARS-CoV-2 in patients with COVID-19 pneumonia.

## Methods

### Study design and participants

This retrospective study was approved by the ethics committee of Zhongnan Hospital of Wuhan University (No. 2020011). All consecutive patients with confirmed COVID-19 admitted to Zhongnan Hospital of Wuhan University from 15th January to 2nd March were enrolled. The exclusion criteria were as follows: (1) patients had negative throat swabs at admission and (2) patients died before negative conversion of swab virus test. Written or oral informed consent was obtained from patients.

### Definitions

COVID-19 was confirmed by detecting SARS-CoV-2 RNA in throat swab samples using a virus nucleic acid detection kit according to the manufacturer's protocol (Shanghai BioGerm Medical Biotechnology Co., Ltd.). Then, all patients were admitted and isolated for treatment within 1 week after symptom onset. During the hospitalisation, each patient had a swab virus test every other day. Negative conversion time of SARS-CoV-2 was defined as the interval between symptom onset and the first of two consecutive negative virus tests.

In severity assessment on admission, serious illness was defined if satisfying at least one of the following items: (i) breathing rate ≥30/min; (ii) pulse oximeter oxygen saturation (SpO_2_) ≤93% at rest and (iii) ratio of partial pressure of arterial oxygen (PaO_2_) to fraction of inspired oxygen (FiO_2_) ≤300 mmHg (1 mmHg = 0.133 kPa). Critical illness was defined if satisfying at least one of the following items: (i) respiratory failure occurred and received mechanical ventilation; (ii) shock and (iii) combined with failure of other organs and received care in the intensive care unit.

### Data collection

A COVID-19 case report form was designed to document primary data regarding demographic, clinical, laboratory, radiological and therapeutic characteristics from electronic medical records. The following information was extracted from each patient: gender, age, medical history, chief complaints and severity assessment on admission, laboratory findings, treatment and negative conversion time of SARS-CoV-2.

### Statistical analysis

Categorical data were described as percentages, and continuous data as median with IQR. Nonparametric comparative test for continuous data and *χ*^2^ test for categorical data were used to compare variables between groups. *P* < 0.05 was considered statistically significant. The significant variables in univariate analysis were put into the multivariate analysis to identify independent risk factors associated with negative conversion time. Receiver operating characteristic (ROC) curve analysis was conducted to evaluate the diagnostic accuracy of each factor. For those reliable factors, an integrated indicator was obtained by a logistics regression model, and its diagnostic power was also evaluated. All statistical analyses were performed using SPSS Statistics version 21.0 software.

## Results

### Baseline characteristics

In total, 169 patients with COVID-19 pneumonia were included in this study. The median age was 51 years (IQR: 36–64), and 83 patients (49.1%) were male. Sixty-four patients (37.9%) had at least one comorbidity, and it was the most common in the cardiovascular/cerebrovascular system (20.1%). Fever (71.0%), fatigue (24.9%), cough (29.0%) and chest distress/shortness of breath (24.9%) were the most common chief complaints. On admission, 20 (11.8%) and 29 (17.2%) patients were categorised into serious and critical illness respectively (Table 1).

**Table 1.** Characteristics of negative conversion time of SARS-CoV-2 from symptom onset in patients with COVID-19 pneumonia

Variable (unit, normal range)	No. (%) or median (IQR)			*P* value
	Total (*n* = 169)	<18 days (*n* = 81)	≥18 days (*n* = 88)	
Male	83 (49.1)	34 (42.0)	49 (55.7)	0.076
Age	51 (36–64)	42 (33–59)	58 (46–66)	0.000
Comorbidities	64 (37.9)	22 (27.2)	42 (47.7)	0.006
Cardiovascular/cerebrovascular	34 (20.1)	10 (12.3)	24 (27.3)	0.015
Respiratory	8 (4.7)	0 (0)	8 (9.1)	0.005
Endocrine	25 (14.8)	8 (9.9)	17 (19.3)	0.085
Nervous	3 (1.8)	1 (1.2)	2 (2.3)	0.612
Urinary	1 (0.6)	0 (0)	1 (1.1)	0.339
Hepatic	9 (5.3)	5 (6.2)	4 (4.5)	0.640
Chief complaints on admission				
Fever	120 (71.0)	56 (69.1)	64 (72.7)	0.610
Fatigue	42 (24.9)	17 (21.0)	25 (28.4)	0.267
Headache	2 (1.2)	2 (2.5)	0 (0)	0.140
Cough	49 (29.0)	16 (19.8)	33 (37.5)	0.011
Chest distress/shortness of breath	42 (24.9)	12 (14.8)	30 (34.1)	0.004
Anorexia	4 (2.4)	2 (2.5)	2 (2.3)	0.934
Diarrhoea	3 (1.8)	1 (1.2)	2 (2.3)	0.612
Severity assessment on admission				
General	120 (71.0)	69 (85.2)	51 (58.0)	0.000
Serious	20 (11.8)	6 (7.4)	14 (15.9)	
Critical	29 (17.2)	6 (7.4)	23 (26.1)	
Blood cytology				
Leucocytes (3.5–9.5 × 10^9^/l)	4.65 (3.34–6.85)	4.06 (3.3–5.78)	5.71 (3.47–9.11)	0.000
Neutrophils (1.8–6.3 × 10^9^/l)	3.2 (2.08–5.35)	2.54 (1.87–3.81)	3.94 (2.31–7.75)	0.000
Platelets (125–350 × 10^9^/l)	178 (134–230)	182 (150–227)	175 (123–234)	0.617
Monocytes (0.1–0.6 × 10^9^/l)	0.4 (0.3–0.5)	0.4 (0.3–0.5)	0.4 (0.3–0.6)	0.846
Lymphocytes (1.1–3.2 × 10^9^/l)	0.81 (0.57–1.07)	0.96 (0.69–1.25)	0.69 (0.46–0.95)	0.261
CD3^+^ (805–4459/μl)	542 (315–883)	692 (378–1044)	481 (298–823)	0.151
CD3^+^CD4^+^ (345–2350/μl)	304 (178–552)	439 (238–620)	240 (142–479)	0.010
CD3^+^CD8^+^ (345–2350/μl)	212 (121–364)	249 (150–413)	201 (100–336)	0.531
CD4/CD8 ratio (0.96–2.05)	2 (1–2)	2 (1–3)	1 (1–2)	0.358
CD19^+^ (240–1317/μl)	121 (56–222)	142 (81–203)	113 (50–232)	0.770
CD16^+^CD56^+^ (210–1514/μl)	136 (56–234)	154 (63–237)	131 (50–231)	0.444
Blood inflammatory indicators				
IFN-*γ* (0.1–18 pg/ml)	0.52 (0.11–1.06)	0.79 (0.40–1.19)	0.13 (0.11–1.06)	0.529
IL-2 (0.1–4.1 pg/ml)	0.56 (0.22–1.32)	0.51 (0.19–1.35)	0.56 (0.22–1.38)	0.590
IL-4 (0.1–3.2 pg/ml)	0.3 (0.1–1.0)	0.11 (0.10–0.54)	0.42 (0.10–1.09)	0.234
IL-6 (0.1–2.9 pg/ml)	16.06 (5.87–34.46)	12.48 (4.9–23.57)	19.9 (7.06–59.99)	0.285
IL-10 (0.1–5.0 pg/ml)	2.24 (1.03–5.55)	2.13 (1.21–4.22)	2.24 (0.95–6.02)	0.346
TNF-*α* (0.1–23.0 pg/ml)	0.1 (0.1–0.1)	0.1 (0.1–0.49)	0.1 (0.1–0.1)	0.659
CRP (0–10 mg/l)	22.2 (4.7–54.3)	17.7 (3.3–44.1)	25.9 (8.8–63.2)	0.120
ESR (0–15 mm/h)	24 (10–41)	19 (8–37)	25 (18–54)	0.050
Blood biochemistry				
ALT (9–50 U/l)	23 (16–33)	22 (14–32)	24 (18–38)	0.169
AST (15–40 U/l)	27 (20–38)	25 (19–32)	31 (21–44)	0.007
ALB (40–55 g/l)	37.2 (32.6–41.1)	39.1 (35.6–42.3)	35.5 (30–38.3)	0.000
GLB (20–30 g/l)	29 (26.9–31.7)	28.4 (26.5–31.1)	29.2 (27.3–32.1)	0.994
ALB/GLB ratio (1.5–2.5)	1.3 (1.06–1.5)	1.39 (1.2–1.54)	1.19 (1.0–1.4)	0.794
GGT (8–57 U/l)	28 (17–46)	22 (15–36)	33 (20–50)	0.209
ALP (30–120 U/l)	66 (54–85)	64 (52–82)	69 (58–85)	0.197
Creatinine (64–104 U/l)	63.5 (52.2–73.7)	60.9 (52.6–72.7)	65 (50.3–76.8)	0.941
Glucose (3.9–6.1 mmol/l)	6.18 (5.26–8.46)	5.92 (5.24–7.37)	6.51 (5.45–10.13)	0.050
LDH (125–243 U/l)	201 (155–281)	189 (142–246)	234 (172–323)	0.368
Creatine kinase (<171 U/l)	89 (53–182)	71 (50–125)	108 (59–203)	0.027
D-dimer (0–500 ng/ml)	242 (122–497)	200 (106–388)	270 (142–678)	0.345
Hospitalised treatment				
Corticosteroid	88 (52.1)	36 (44.4)	52 (59.1)	0.057
Mechanical ventilation	28 (16.6)	6 (7.4)	22 (25.0)	0.002
Virus negative conversion time	18 (11–25)	11 (8–13)	25 (21–29)	0.000

COVID-19, coronavirus disease 2019; No., number; IQR, interquartile range; IL, interleukin; TNF, tumour necrosis factor; CRP, C-reactive protein; ESR, erythrocyte sedimentation rate; ALT, alanine aminotransferase; AST, aspartate aminotransferase; ALB, albumin; GLB, globulin; GGT, glutamyltranspetidase; ALP, alkaline phosphatase; LDH, lactate dehydrogenase.

**Fig. 1.**
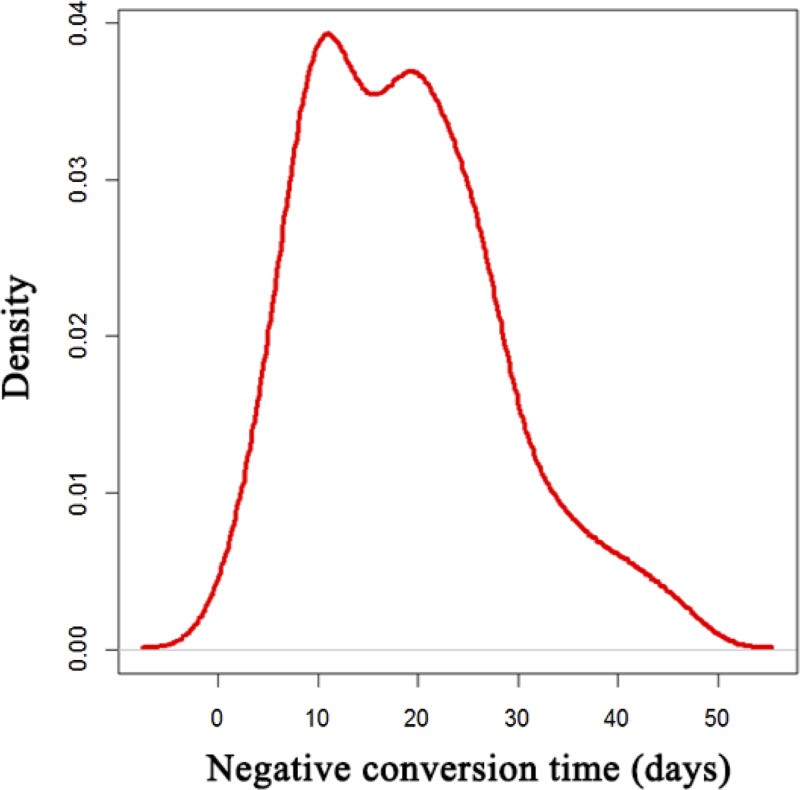
Distribution of SARS-CoV-2 negative conversion time among the 169 COVID-19 patients.

During the hospitalisation, 88 patients (52.1%) received intravenous corticosteroid (methylprednisolone, 20–120 mg daily for 3–15 days), and 28 (16.6%) with mechanical ventilation. The median negative conversion time of SARS-CoV-2 was 18 days (IQR: 11–25) from symptom onset (Fig. 1). In total, 42.5% of general cases, 70% of serious cases and 79% of critical cases still tested positive at or beyond day 18 of symptom onset.

### Univariate analysis of risk factors for delayed virus negative conversion

The patients were divided into two groups according to the median negative conversion time. Compared with the patients with short-term negative conversion, those with long-term conversion had an older age, higher incidence of comorbidities, especially cardiovascular/cerebrovascular and respiratory systems, chief complaints of cough and chest distress/breath shortness and severer disease assessment on admission. All patients had a comprehensive laboratory examination within 1 week after symptom onset. The patients with long-term conversion had a higher level of leucocytes, neutrophils, aspartate aminotransferase (AST), creatine kinase and erythrocyte sedimentation rate (ESR), and a lower level of CD3^+^CD4^+^ lymphocytes and albumin. During the hospitalisation, the patients with long-term negative conversion were more likely to receive mechanical ventilation.

### Multivariate analysis and ROC analysis

The significant variables in univariate analysis were put into the multivariate analysis to identify independent risk factors associated with delayed negative conversion. Finally, cough, leucocytes, neutrophils and ESR were positively correlated with delayed virus negative conversion, and CD3^+^CD4^+^ lymphocytes were negatively correlated (Table 2).

**Table 2.** Multivariate analysis of factors associated with negative conversion time of SARC-CoV-2 from symptom onset in patients with COVID-19 pneumonia

	*B*	s.e.	Wald's	*P* value	OR (95% CI)
Male	−0.134	0.896	0.022	0.881	0.875 (0.151–5.064)
Comorbidities	1.446	1.094	1.746	0.186	4.244 (0.497–36.214)
Cardiovascular/cerebrovascular	0.210	1.179	0.032	0.859	1.233 (0.122–12.423)
Respiratory	19.517	18011.978	0.000	0.999	na
Cough	2.030	0.879	5.333	0.021	7.617 (1.359–42.676)
Chest distress/breath shortness	−0.416	1.150	0.131	0.718	0.660 (0.069–6.290)
Severity assessment on admission	1.600	1.394	1.319	0.251	4.955 (0.323–76.069)
Leucocytes	2.368	0.934	6.432	0.011	10.677 (1.712–66.570)
Neutrophils	−2.332	0.955	5.960	0.015	0.097 (0.015–0.631)
CD3^+^CD4^+^ lymphocytes	−0.009	0.003	9.454	0.002	0.991 (0.985–0.997)
AST	−0.036	0.036	1.050	0.306	0.964 (0.899–1.034)
Albumin	−0.010	0.084	0.016	0.900	0.990 (0.840–1.166)
Creatine kinase	0.004	0.004	1.239	0.266	1.004 (0.997–1.011)
ESR	−0.036	0.018	3.927	0.048	0.964 (0.930–1.000)
Mechanical ventilation	−3.920	2.850	1.892	0.169	0.020 (0.000–5.290)

COVID-19, coronavirus disease 2019; na, not available; OR, odds ratio; CI, confidence interval; AST, aspartate aminotransferase; ESR, erythrocyte sedimentation rate.

**Fig. 2.**
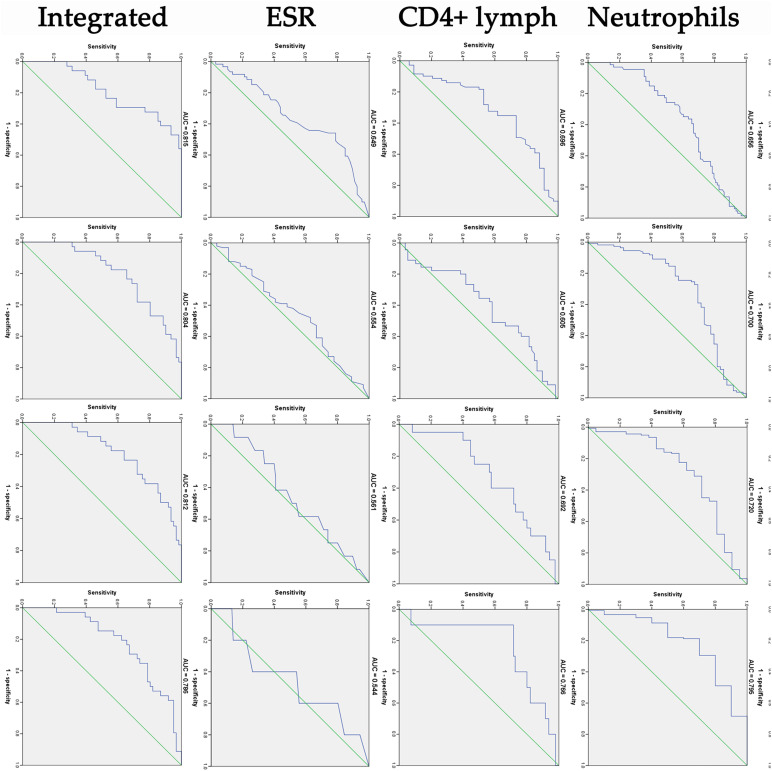
ROC curve analysis of cough, leucocytes, neutrophils, CD3^+^CD4 lymphocytes, ESR and integrated indicator in predicting the negative conversion of SARS-CoV-2 within 2, 3, 4 and 5 weeks from symptom onset. AUC, area under ROC curve.

In ROC analysis, leucocytes, neutrophils and CD3^+^CD4^+^ lymphocytes showed a relatively higher diagnostic accuracy in predicting the virus negative conversion time (Fig. 2). The integrated indicator comprising these three factors had a good performance in predicting the negative conversion within 2 weeks (area under ROC curve (AUC) = 0.815), 3 weeks (AUC = 0.804), 4 weeks (AUC = 0.812) and 5 weeks (AUC = 0.786). To validate the robustness of the integrated indicator, we also randomly selected three factors (red blood cells, gender and tumour necrosis factor-*α*) to obtain a new integrated factor, and evaluated its predictive ability according to the same method. Finally, the new integrated indicator showed a poor predictive ability (AUC = 0.583) for 2 weeks, significantly lower than the previous integrated indicator.

## Discussion

COVID-19 is mainly transmitted through both respiratory droplets and close contact from person-to-person. SARS-CoV-2 RNA can be detected in throat swab samples, sputum, alveolar lavage fluid, blood and faeces of patients with COVID-19 [11, 12]. Several studies suggested that the virus positive rate of lower respiratory tract specimens, especially bronchoalveolar lavage fluid, was higher than that of upper respiratory specimens in COVID-19 patients [13–15]. Bronchoalveolar lavage fluid specimens showed the highest positive rates (93%), followed by sputum (72%), nasal swabs (63%) and pharyngeal swabs (32%) [11]. However, lower respiratory sampling involves exposure risk and greater technical difficulty. Because of its convenience, throat swab testing is the most common method for screening COVID-19. It has limitations and possible false-negative results. In order to improve the accuracy of detection, repeated testing is needed in clinical practice. In this study, each patient had a swab virus test every other day during their hospitalisation.

In this retrospective cohort study, the median negative conversion time of SARS-CoV-2 was 18 days (IQR: 11–25). This result was similar to that of another study, in which median duration of viral shedding was 17 days (IQR: 12–21) [16]. Viral load kinetics of SARS-CoV-2 infection has been described in a Korean study [17]. In one patient, the virus was detected from upper respiratory tract specimens on day 3 of symptom onset. The virus load increased, peaked on day 7, and then gradually decreased. Finally, the assay became undetectable for 2 consecutive days from day 15.

In our study, all the patients were divided into two groups according to 18 days of negative conversion time to identify the risk factors associated with delayed negative conversion. Compared with the patients with short-term negative conversion, those with long-term conversion had an older age, higher incidence of comorbidities, chief complaints of cough and severer illness on admission. COVID-19 patients with older age were more likely to progress to severe disease and had a higher mortality rate [18–20]. A study of the viral dynamics showed that patients with severe COVID-19 tended to have a high viral load and a long virus-shedding period [21]. All severe cases still tested positive at or beyond day 10 of symptom onset. By contrast, 90% of mild cases repeatedly tested negative by day 10 of symptom onset. In this study, 42.5% of general cases, 70% of serious cases and 79% of critical cases still tested positive at or beyond day 18 of symptom onset. These differences may be related to the population composition and region of the study. A previous study showed that SARS-CoV-2 RNA was more readily detected in induced sputum than in throat swabs of convalescent COVID-19 patients [22]. In this study, COVID-19 patients with cough symptoms were more likely to have long-term conversion. We speculated that the patient expelled the virus from the lower respiratory tract by coughing, and the positive rate of detection would be higher, thus resulting in the prolonged virus negative conversion.

In terms of laboratory tests, we noted that most of COVID-19 patients presented lymphopoenia, decreased level of lymphocyte subsets and elevated levels of infection related biomarkers (including IL-6, ESR and lactate dehydrogenase), which was consistent with recent reports [23, 24]. More interestingly, a higher number of leucocytes and neutrophils were found in the long-term negative conversion group compared to the short-term negative conversion group. Leucocytes and neutrophils were well-known markers of systemic inflammation, which had been studied as a predictor of bacterial infection [25]. Previous studies had shown that neutrophilia was the risk factor related to the development of ARDS and progression from ARDS to death in COVID-19 patients [18, 26]. In this study, neutrophilia was the risk factor related to the prolonged virus negative conversion. Neutrophils were the main source of cytokines. Excessive neutrophils contributed to acute lung damage and cytokine storm which might be related to the delay of virus clearance.

In this study, we found that a decreased number of CD3^+^CD4^+^ lymphocytes was another risk factor for delayed negative conversion of SARS-CoV-2 in COVID-19 patients. T lymphocytes, especially CD3^+^CD4^+^ lymphocytes, played a significant antiviral role in the combat against MERS or SARS [27, 28]. COVID-19 might damage lymphocytes, especially T lymphocytes, and the immune system was impaired during the period of disease. CD3^+^CD4^+^ lymphocytes were crucial for maintaining the efficient immune response. A study about immune response of COVID-19 suggested that both CD3^+^CD4^+^ lymphocytes and CD3^+^CD8^+^ lymphocytes were lower than normal levels in COVID-19 patients, even much lower in severe cases [29]. In another of our recent study [30], we continuously observed the changes of lymphocytes, which gradually increased with the recovery of the disease, and the virus tests turned negative. Thus, delayed elimination of the virus might be due to the serious disturbance of immune system. The specific immunological mechanism needed further study.

Furthermore, in ROC analysis of this study, leucocytes, neutrophils and CD3^+^CD4^+^ lymphocytes showed a relatively higher diagnostic accuracy in predicting the virus negative conversion time. The integrated indicator comprising these three factors had a good performance in predicting the negative conversion within 2, 3, 4 and 5 weeks. In areas with limited SARS-CoV-2 RNA testing conditions, isolation time of COVID-19 could be roughly determined according to these indicators to reduce virus transmission and medical burden.

In our research, it was worth mentioning that corticosteroid therapy in COVID-19 patients was not associated with delayed SARS-CoV-2 RNA clearance. Corticosteroid therapy was commonly used in critical patients and had always been controversial in the treatment of viral pneumonia. The high-dose systemic corticosteroid therapy was associated not only with increased blood viral loads but also with adverse effects and increased mortality in patients infected with SARS-CoV or MERS-CoV [31–33]. However, a recent study showed that treatment with corticosteroid might be beneficial for COVID-19 patients who developed ARDS [26]. Further studies are needed to confirm the role of corticosteroid in the treatment of COVID-19.

Our study has some limitations. First, there might be a few false-negative results of SARS-CoV-2 RNA tests. The polymerase chain reaction (PCR) test is the most widespread and most accurate diagnostic test for COVID-19. The overall positivity of RT-PCR for SARS-CoV-2 was around 30–40% [34]. The risk of false-negative test results depends on the timing and quality of the test samples, the quality of detection reagents and the accuracy of the laboratory analysis. Moreover, selection bias might have occurred because this was a retrospective single-centre study, and further prospective studies were needed.

## Conclusion

Our study showed that the median time of SARS-CoV-2 negative conversion was 18 days (IQR: 11–25) in patients with COVID-19 pneumonia. The current policy of 14 days of mandatory quarantine for everyone might be too conservative. Longer quarantine periods might be more justified for COVID-19 patients with cough, higher levels of leucocytes, neutrophils and ESR and lower levels of CD3^+^CD4^+^ lymphocytes.

## Data Availability

The data that support the findings of this study are available from the corresponding author and can be obtained by request at E-mail: znact1936@126.com.
